# Hypoxia-Induced Mitogenic Factor: A Multifunctional Protein Involved in Health and Disease

**DOI:** 10.3389/fcell.2021.691774

**Published:** 2021-07-15

**Authors:** Moyang Lv, Wenjuan Liu

**Affiliations:** ^1^Department of Gastroenterology, Xinqiao Hospital, Third Military Medical University, Chongqing, China; ^2^Department of Pathophysiology, Health Science Center, Shenzhen University, Shenzhen, China

**Keywords:** hypoxia-induced mitogenic factor, mitogenesis, angiogenesis, proinflammation, vasoconstriction

## Abstract

Hypoxia-induced mitogenic factor (HIMF), also known as resistin-like molecule α (RELMα) or found in inflammatory zone 1 (FIZZ1) is a member of the RELM protein family expressed in mice. It is involved in a plethora of physiological processes, including mitogenesis, angiogenesis, inflammation, and vasoconstriction. HIMF expression can be stimulated under pathological conditions and this plays a critical role in pulmonary, cardiovascular and metabolic disorders. The present review summarizes the molecular characteristics, and the physiological and pathological roles of HIMF in normal and diseased conditions. The potential clinical significance of these findings for human is also discussed.

## Introduction

Hypoxia-induced mitogenic factor (HIMF), also known as found in inflammatory zone 1 (FIZZ1) or resistin-like molecule α (RELMα), is a pro-inflammatory cytokine in mice. HIMF expression is markedly increased in the hyperplastic bronchial epithelium of mice with allergic pulmonary inflammation (Holcomb et al., 2000). Three years later, Teng et al. (2003) also reported a novel role for this molecule in the pathogenesis of hypoxia-induced pulmonary hypertension (PH). Hypoxia was found to induce HIMF expression, and the protein was upregulated in a murine chronic hypoxia model of pulmonary hypertension. The authors renamed this gene HIMF, as the recombinant protein stimulated the proliferation of rat pulmonary microvascular smooth muscle cells (PSMCs). Following investigations using a chronic hypoxia-induced PH mouse model uncovered a pluoripotent array of pathological roles played by HIMF, including in angiogenesis, vasoconstriction, inflammation and fibrosis. Together, these properties endow HIMF with a critical function in pulmonary vascular remodeling and, thus, in the development of PH after hypoxia. Notably, HIMF has also been linked to both cell survival and cell death, depending on the dose and cellular context. For example, despite the harmful effects of increased HIMF expression in lung disease, particularly in PH, basal expression of HIMF is required for normal lung development.

In addition to its involvement in pulmonary disease, a number of studies have demonstrated that HIMF also participates in the development of cardiovascular diseases and metabolic disorders in mice. Furthermore, recent studies by our group have uncovered a novel role for HIMF in the pathogenesis of pressure overload-induced cardiac hypertrophy and fibrosis (Kumar et al., 2018, 2019). These findings are of particular note because the two human homologs of HIMF, RELMβ, and resistin (hRETN), are also associated with cardiovascular diseases. For example, there is a positive correlation between resistin plasma levels and the incidence of cardiovascular events in patients with cardiovascular diseases (Zhang et al., 2011). Furthermore, studies in mice have revealed a causal relationship between the RELMβ expression and the pathogenesis of atherosclerosis (Kushiyama et al., 2013). Therefore, HIMF (and, by extension, its human homologs) may represent potential, clinically significant biomarkers or therapeutic targets for the diagnosis, prognosis and treatment of cardiovascular diseases. The pro-inflammatory properties of HIMF are of particular interest, as these contribute to metabolic complications which are closely associated with chronic low-grade inflammation. Similar to its function in lung tissue, HIMF regulates glucose and energetic metabolism in dose and cell-dependent manners. Increased HIMF expression results in metabolic disturbance, but basal levels of HIMF expression by a certain group of immune cells (CD301b^+^ mononuclear phagocytes) is required for the maintenance of metabolic homeostasis (Kumamoto et al., 2016).

This review summarizes the molecular characteristics of HIMF, including its molecular structure, tissue-specific distribution, transcription mechanisms and receptors. We primarily focus on the pluripotent physiological and pathological functions of HIMF, particularly in the pathogenesis of pulmonary, cardiovascular and metabolic disorders, alongside, the respective downstream signaling pathways. The potential clinical significance of HIMF is also discussed.

## Molecular Characterizations

### Structure

HIMF (RELMα, also known as FIZZ1) is a 9.4 kDa cysteine-rich secretory cytokine that belongs to the FIZZ/RELM family, which also includes RELMβ (FIZZ2) and RELMγ. RELMα, and FIZZ1, as well as RELMβ and FIZZ2 were discovered independently by separate, unrelated labs as different functional proteins, but finally proved to be the same. The RELM proteins share a similar cysteine composition and other signature features with resistin (known as FIZZ3 in mice). The RELM family proteins typically range from 105 to 114 amino acids in length and are composed of three domains: an N-terminal sequence containing a secretory signal peptide, a variable middle portion, and a unique, highly conserved C-terminal signature sequence that contains 10 cysteine residues (Banerjee and Lazar, 2001). Members of the RELM family show strong interspecies and intraspecies homology, especially at this cysteine-rich C-terminus.

### Tissue-Specific Distribution

The expression of HIMF, as with other members of the RELM family, is uneven across different types of tissue. For example, expression of HIMF (FIZZ1) is 10-fold higher in murine lung tissue compared with heart tissue or skeletal muscle (Holcomb et al., 2000). Alongside the lung and heart, HIMF is also expressed in the tongue, but its expression is highest in adipose tissue (Steppan et al., 2001). In addition, HIMF has also been found to mediate myeloid cell chemotaxis (Su et al., 2007). Meanwhile, RELMβ (FIZZ2) is exclusively expressed in the gastrointestinal tract, specifically in actively replicating crypt endothelium of the colon and small bowel (Hogan et al., 2006), similar to the findings by Holcomb. RELMγ has been found to be expressed in hematopoietic tissues and lung in rodents (Gerstmayer et al., 2003), and FIZZ3 is expressed in white adipocytes throughout the body (Holcomb et al., 2000).

In addition to these initial findings, RELM proteins may also be expressed in other tissues under pathological conditions or during development. For example, HIMF and other RELM proteins are expressed in the murine liver during helminth-induced Th2-type immune responses (Pesce et al., 2009). HIMF is also expressed by macrophages, and increased HIMF levels can be used as a marker for alternatively activated (M2) macrophages in mice. RELMβb and resistin are homologs of HIMF, and the expression patterns of RELMβ in the human lung are similar to those of HIMF in mice. Human resistin is also expressed in myeloid cells, especially macrophages, with a similar expression pattern to murine HIMF. Advances in protein quantification techniques are also revealing novel expression sites for RELM proteins, with hResistin expression recently being characterized across normal human tissues using a newly developed monoclonal antibody (Lin et al., 2020). hResistin was found to be principally localized in the cytoplasmic granules of macrophages, which are present in the interstitial space of the majority of human tissues. Therefore, the functions of HIMF in rodents may indicate potential roles of human RELMβ and resistin depending on the induction site and the cellular source of the protein.

### Transcription Mechanisms

The mechanisms involved in the induction of HIMF transcription remain to be fully characterized, and may vary between physiological and pathological conditions. However, its expression is altered during development. For example, HIMF expression is upregulated in embryonic mouse lung tissue, and is involved in normal lung development. The transcription factor Ets-1, which is also expressed in the developing mouse lung, has been found to contribute to developmental expression of HIMF (Li et al., 2007). In this study, Ets-1 was found to increase HIMF promoter luciferase activity in a heterogenous expression system, and chromatin immunoprecipitation (ChIP) assays revealed that Ets-1 bound to the HIMF promoter region in embryonic day (E) 20 lung tissues. Ets-1 is also known to participate in the transcriptional activation of vascular endothelial growth factor receptor-2 (Flk-1), in coordination with hypoxia-inducible factor 2α (HIF-2α), during vascular development and angiogenesis (Elvert et al., 2003). HIF-2α expression is co-localized with HIMF in the developing airway epithelial cells and alveolar type II cells, suggesting that it may also induce HIMF expression during lung development (Wagner et al., 2004).

Furthermore, signal transducers and activators of transcription 6 (STAT6) and C/EBP have been suggested to mediate HIMF induction during the Th2 inflammatory response (Yamaji-Kegan et al., 2010). Cellular experiments demonstrated that HIMF promoter reporter gene constructs respond to Th2-cytokines, IL-4, and IL-13 stimulation. In addition, the promoter region of the HIMF gene was found to contain functional binding sites for signal transducers and activators of transcription 6 (STAT6) and C/EBP. Point mutations in the STAT6 or the C/EBP sites led to the loss of cytokine responsiveness, indicating that Th2-related HIMF induction is orchestrated by the coordinated action of STAT6 and C/EBP. The involvement of STAT6 in the mediation of HIMF production has also been confirmed in a murine model of acute pulmonary inflammation, with HIMF found to be upregulated 6 h after antigen challenge. Notably, this effect was abolished by STAT6 gene ablation.

The potential role of hypoxia inducible factor-1α (HIF-1α) in HIMF induction, particularly in hypoxia-induced PH, has also generated a great deal of attention. HIF-1α is also activated during hypoxia, and serves a critical function in both hypoxic inflammation and Th2 immune activation in the lung. Previous experiments in HIF-1α heterozygous null (HIF-1α^+/–^) mice also found that HIMF induced HIF-1α expression, and HIMF-induced PH was significantly diminished in HIF-1α^+/–^ mice (Johns et al., 2016). In addition, recent work by our group revealed that HIMF increases HIF-1α expression and cardiomyocyte hypertrophy, but HIF-1α activation has no impact on HIMF expression (Kumar et al., 2018). Taken together, these results suggest that HIF-1α is a critical downstream mediator of HIMF-induced PH and cardiac hypertrophy, rather than an upstream transcription factor controlling HIMF expression.

### Receptors

Although a number of signaling pathways are activated by HIMF, the functional receptors for HIMF, resistin and other RELM proteins, remain unclear. Previous research has determined that HIMF induces the release of intracellular Ca^2+^ in pulmonary artery smooth muscle cells through the PLC-IP3 pathway (Fan et al., 2009). This suggests that HIMF may act as a ligand of G protein coupled receptors (GPCRs), the activation of which stimulates PLC-IP3 signaling. This previous study further revealed that the HIMF-induced Ca^2+^ response was attenuated by the tyrosine kinase inhibitor genistein. In addition, the pattern of Ca^2+^ release was altered by Gα_q/__11_ knockdown, from sustained oscillatory Ca^2+^ transients with prolonged plateaus to a series of short Ca^2+^ transients. However, this effect was not elicited by Gα_*i*_ or Gα_*s*_ knockdown. These results led the authors to conclude that Gα_q/__11_ protein-coupled receptors and a receptor tyrosine kinase are critical to HIMF-induced Ca^2+^ signaling. A more recent study from the same group demonstrated that Bruton’s tyrosine kinase (BTK) is a binding partner for HIMF (Lin et al., 2019a), providing further evidence to suggest that HIMF functions via intracellular receptor tyrosine phosphorylation. However, the exact GPCRs involved remain unknown.

A recent study has reported that extracellular calcium-sensing receptor (CaSR) acts as a receptor for intracellular HIMF (Zeng et al., 2017). Using a yeast 2-hybrid assay, the authors found that HIMF bound to the intracellular domain of CaSR, increasing the activity of the receptor. In turn, this mediated the hypoxia-induced proliferation of PSMCs, pulmonary vascular remodeling, and consequent pulmonary hypertension. However, CaSR appears to be a non-classical receptor for HIMF, mediating intracellular HIMF signaling only. Although the classical HIMF receptor remains to be identified, it is interesting that a synthesized, membrane-permeable peptide targeting the intracellular binding domain of CaSR for HIMF attenuated the development of hypoxia-induced PH.

## Pluoripotent Effects and Associated Signaling Pathways

### Mitogenesis

**FIGURE 1:**
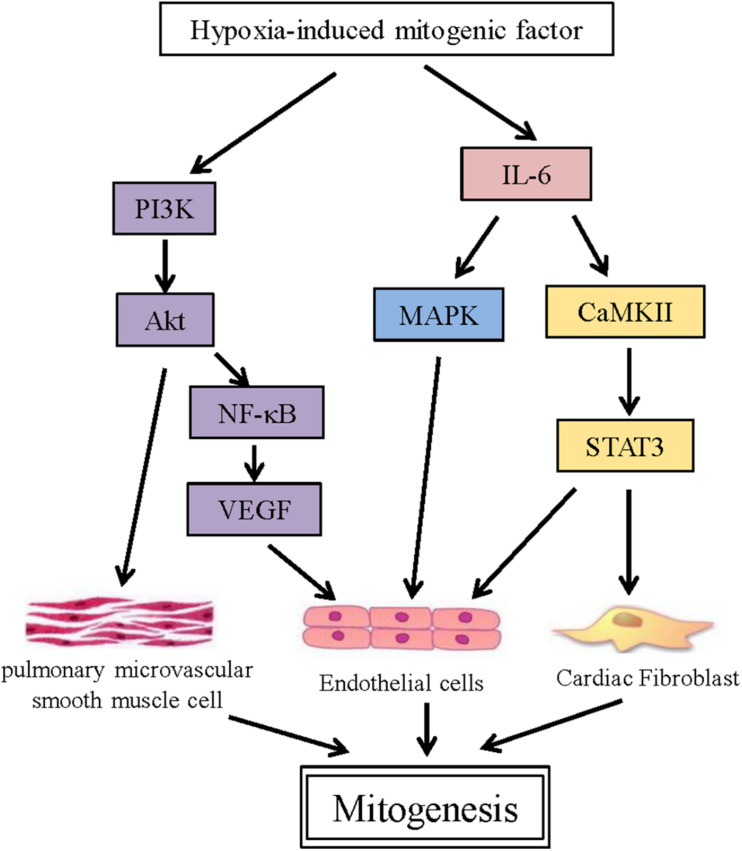
HIMF links multiple mitogenesis-associated pathways. Schematic representing the direct target genes involved in various signaling pathways associated with mitogensis that are induced by HIMF.

One of the most prominent functions of HIMF is its involvement in mitogenesis. Indeed, it was initially termed RELMα, but was renamed after it was found to induce PSMC proliferation (Teng et al., 2003). Subsequent studies demonstrated that HIMF can also induce the proliferation of endothelial and fibroblast cells. The activation of the phosphatidylinositol 3-kinase (PI3K)/Akt pathway is critical during PSMC proliferation, with the inhibition of PI3K significantly suppressing Akt phosphorylation and PSMC proliferation. The PI3K/Akt pathway also contributes to the proliferation of HIMF-induced endothelial cells (ECs), by inducing the production of vascular endothelial growth factor (VEGF) through NF–κB (Tong et al., 2006a; Yamaji-Kegan et al., 2006). In addition, the mitogen-activated protein kinase (MAPK) pathway was also found to be involved in HIMF-induced EC proliferation, with the ERK inhibitor U0126 significantly inhibiting EC proliferation. A recent study by our group demonstrated that HIMF also stimulates cardiac fibroblast (CF) proliferation in a dose-dependent manner (Kumar et al., 2019). Here, IL-6 plays a key role, activating the MAPK and calcium/calmodulin-dependent protein kinase II (CaMKII)-STAT3 pathways (Figure 1).

### Angiogenesis

Angiogenesis involves a cascade of intricately regulated processes, including the dissolvement of the basement membrane and extracellular matrix, EC migration, and subsequent EC proliferation and vascular sprouting (Li et al., 2013). The stimulation of angiogenic growth factors, including VEGFs and fibroblast growth factors (FGFs), plays an important role in this process (Li et al., 2013). HIMF is also strongly associated with angiogenesis. Previous studies have shown that HIMF stimulates VEGF production in vascular ECs, and promotes the proliferation and migration of ECs and vascular sprouting (Tong et al., 2006c; Yamaji-Kegan et al., 2006). Furthermore, suppressing VEGF receptor-2 (VEGFR2) significantly inhibited HIMF-induced angiogenesis. HIMF has also been found to increase the production of Flk-1, which contributes to pulmonary angiogenesis (Tong et al., 2006b). Activation of the PI-3K/Akt-NF-κB signaling pathway has been suggested to mediate the induction of VEGF and Flk-1 expression (Tong et al., 2006a,b). Notably, in addition to targeting endothelial cells directly, HIMF has also been shown to stimulate angiogenesis through the activation of myoblasts (Su et al., 2017). HIMF increases IL-18 production through the PDK1/PI3K/Akt signaling pathway in myoblasts, which in turn promotes tube formation of the endothelial progenitor cells.

Angiogenesis is closely linked to inflammation. Pro-inflammatory cells, particularly macrophages, not only release large quantities of angiogenic factors under pathological conditions, but also indirectly promote angiogenesis through releasing inflammatory factors that recruit endothelial progenitor cells, which also promote vascular formation. HIMF has been found to recruit CD68-positive cells in mouse lungs and to stimulate the production of monocyte chemotactic protein-1 (MCP-1) and stromal cell-derived factor-1 (SDF-1), which are both angiogenesis-related inflammatory factors (Yamaji-Kegan et al., 2006). These effects were suppressed by treatment with a VEGFR2 inhibitor, providing further evidence to support a causal link between HIMF activity and VEGF-associated angiogenesis.

### Inflammation

HIMF is a well-known marker of activated M2 anti-inflammatory macrophage. One of the most abundant neuropeptides in lung, calcitonin gene-related peptide (CGRP), has been found to attenuate lipopolysaccharide (LPS)-induced acute lung injury in rats. CGRP significantly reduced LPS-induced NLRP3 activation and increased the expression of HIMF, which was induced by IL-4 in macrophages (Duan et al., 2017). Similar results were reported in the liver of human cholestatic patients and bile duct-ligated mice. The activation of the NLRP3 inflammasome and increased numbers of M2 anti-inflammatory macrophages, as evidenced by increased HIMF expression, aggravated hepatic injury (Cai et al., 2020). Meanwhile, the inhibition of inflammation and induction of M2 macrophage polarization via the miR-223/TRAF6/NF-κB axis alleviated viral myocarditis (Xue et al., 2020).

Previous studies have shown that HIMF induces the production and release of an array of pro-inflammatory factors, including IL-4 (Yamaji-Kegan et al., 2014), IL-6 (Johns et al., 2016), IL-18 (Su et al., 2017), TNF-α (Song et al., 2012), HMGB1 (Lin et al., 2019a,b), vascular adhesion molecule-1 VCAM-1 (Tong et al., 2006d), MCP-1 and SDF-1 (Yamaji-Kegan et al., 2010). A single injection of recombinant HIMF induced IL-4 production and lung injury in mice, while ablation of IL-4 abolished the recruitment of macrophages to the lung and the pulmonary vascular inflammation caused by HIMF (Yamaji-Kegan et al., 2014). In hypoxia-induced PH, HIMF induced the recruitment of macrophages and α-SMA-production cells, and increased IL-6 production via HIF-1α activation (Johns et al., 2016). HIMF also upregulates VCAM-1 expression and mononuclear cell sequestration to the lung parenchyma in bacterial lipopolysaccharide (LPS)-induced acute lung injury, increasing its severity (Tong et al., 2006d). Furthermore, HIMF mediates EC-smooth muscle cell crosstalk, affecting HMGB1-RAGE signaling (Lin et al., 2019a), and induces macrophage-specific HMGB1/RAGE expression. This, in turn, increases the apoptosis-resistant proliferation of human pulmonary artery smooth muscle cells during pulmonary vascular remodeling (Lin et al., 2019b). The induction of the pro-inflammatory factors MCP-1 and SDF-1 by HIMF also results in vascular remodeling in PH. Meanwhile, in the heart, HIMF also stimulates IL-6 production in cardiomyocytes and cardiac fibroblasts via the activation of the MAPK and CaMKII–STAT3 pathways (Kumar et al., 2019).

Interestingly, HIMF appears to regulate T helper type 2 (Th2)-induced inflammation in a different fashion. HIMF expression is induced by the Th2 cytokines IL-4 and IL-13, and serves as a biomarker for the transformation of alternatively activated macrophages (AAMacs). The activation of AAMacs is the hallmark of several inflammatory conditions associated with parasite infection, allergy, diabetes and cancer (Nair et al., 2009). In a *Schistosoma mansoni (Sm)* eggs-induced mouse model of Th2 cytokine-dependent lung inflammation, HIMF suppressed Th2 cytokine-mediated pulmonary inflammation, and ablation of HIMF in mice exacerbated lung inflammation (Nair et al., 2009; Pesce et al., 2009). The effect of HIMF on the suppression of helminth-induced Th2-type immunity also occurs in other organs, such as the liver (Pesce et al., 2009). The underlying mechanism is related to the ability of HIMF inhibiting macrophage and CD4^+^ cells-mediated Th2 cytokine production in a Bruton’s tyrosine kinase-dependent manner (Nair et al., 2009).

### Vasoconstriction

In addition to the aforementioned processes, HIMF is also involved in vasoconstriction of the pulmonary artery. For example, intravenous injection of HIMF is known to increase pulmonary arterial pressure and pulmonary vascular resistance in mice. Surprisingly, the constrictive effect of HIMF is even more potent than either endothelin-1 or angiotensin II (Teng et al., 2003). This effect has been attributed to regulation of the Ca^2+^ signal by HIMF, with recombinant murine HIMF increasing the intracellular Ca^2+^ concentration in a sustained, oscillatory manner in human pulmonary artery smooth muscle cells (SMCs). Notably, the Ca^2+^ increase is related to IP_3_-mediated intracellular Ca^2+^ release, but not extracellular Ca^2+^ influx. Inhibition of PLC-IP_3_ and tyrosine kinase abolished HIMF-induced Ca^2+^ signaling, while knockdown of Gα_q/__11_ expression and ryanodine pre-treatment altered the pattern of Ca^2+^ release (Fan et al., 2009). Taken together, this suggests that HIMF stimulates intracellular Ca^2+^ release in human pulmonary artery SMCs through the PLC signaling pathway, in an IP3- and tyrosine phosphorylation-dependent manner. Furthermore, Gα_q/__11_ protein-coupled receptors and ryanodine receptors are also involved in Ca^2+^ regulation.

However, whether HIMF exerts similar effects on systemic artery SMCs, and the potential roles of HIMF in regulation of blood pressure, remain to be fully elucidated and warrant further study.

## HIMF and Diseases

### Pulmonary Diseases

#### Pulmonary Hypertension (PH)

**FIGURE 2:**
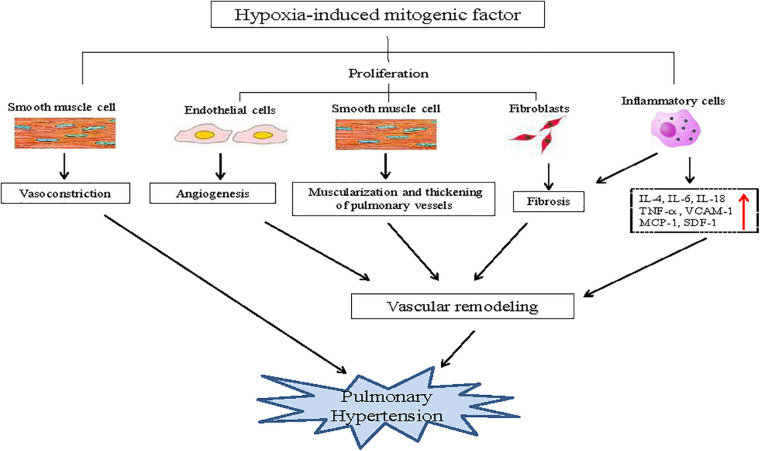
Role of HIMF in hypoxia-induced pulmonary hypertension. HIMF induces pulmonary vasoconstriction, which plays a critical role in pulmonary hypertension. Simultaneously, HIMF also induces the proliferation of pulmonary arterial endothelial cells, smooth muscle cells and fibroblasts, which leads to angiogenesis, muscularization, the thickening of small pulmonary vessels, fibrosis, and subsequent vascular remodeling. Furthermore, HIMF induces inflammation in the lungs, which also promotes vascular remodeling and lung fibrosis, contributing to the pathological process of pulmonary hypertension.

The most well-studied disease associated with HIMF is hypoxia-induced PH; a disease characterized by the progressive elevation of hypoxia-induced pulmonary vascular resistance, the development of right ventricular failure, and ultimately death. The elevation of vascular resistance is primarily attributed to pulmonary artery constriction and vascular remodeling in response to hypoxia. Vascular remodeling involves complicated pathological processes that include angiogenesis, muscularization, the thickening of small pulmonary vessels, inflammation, and fibrosis. HIMF is upregulated in the pulmonary vasculature, bronchial epithelial cells, and type II pneumocytes in hypoxia-induced PH (Teng et al., 2003). HIMF is also known to increase pulmonary vasoconstriction, and has a critical role in each pathological process of vascular remodeling. It enhances the angiogenic capacity of pulmonary myoblasts and the tubule formation of progenitor endothelial cells (Su et al., 2017). Furthermore, HIMF also induces the proliferation of pulmonary SMCs and fibroblast (FCs), as well as the differentiation of FCs, leading to muscularization, thickening of small pulmonary vessels, and lung fibrosis (Teng et al., 2003). In addition, HIMF induces lung inflammation, which promotes vascular remodeling and lung fibrosis (Liu et al., 2009; Angelini et al., 2013; Figure 2).

The human homolog of HIMF, RELMβ, has been shown to be upregulated in the lung tissue of patients with scleroderma-associated pulmonary hypertension (Angelini et al., 2009). RELMβ is primarily localized in the endothelium and vascular smooth muscle of remodeled vessels and in plexiform lesions, macrophages, T cells, and myofibroblast-like cells; similar to HIMF in hypoxia-induced PH in mice. Thus, clarifying the role of HIMF in the development of PH in mice may aid our understanding of the function of human RELMβ in the development of scleroderma-associated PH.

#### Allergic Asthma

HIMF was initially described as present in the bronchoalveolar lavage fluid in a murine allergic pulmonary inflammation model (Holcomb et al., 2000). HIMF production in antigen-challenged lungs has been suggested to be driven by either IL-4 or IL-13, involving transcription factors STAT6 and C/EBP (Stutz et al., 2003; Yamaji-Kegan et al., 2010). Since the activation of STAT6 signaling has a critical role in allergic pulmonary inflammation, the STAT6-dependent induction of HIMF in the alveolar epithelium during pulmonary inflammation and fibrosis suggests that HIMF is involved in asthma pathogenesis. Supporting this, HIMF expression has since been found to be increased in OVA-induced inflammation (Fan et al., 2015). Furthermore, another previous study demonstrated that HIMF inhibited the nerve growth factor (NGF)-mediated survival of rat embryonic dorsal root ganglion (DRG) neurons, in addition to suppressing NGF-induced CGRP gene expression in adult rat DRG neurons (Holcomb et al., 2000). HIMF may modulate the function of neurons innervating the bronchial tree, and thus alter the local tissue response to allergic pulmonary inflammation. Further studies to clarify the role of HIMF in the inflammatory response in allergic asthma are clearly warranted.

### Cardiovascular Diseases

#### Cardiac Hypertrophy and Heart Failure

A number of recent studies have demonstrated that HIMF has a critical role in the development of pressure overload-induced cardiac hypertrophy. For example, HIMF is upregulated in both a phenylephrine (PE)-stimulated cell model of cardiomyocyte hypertrophy, and a transverse aortic constriction (TAC)-induced mouse model of cardiac hypertrophy (Kumar et al., 2018). In this study, cardiomyocyte hypertrophy was induced by HIMF overexpression in cardiomyocytes, while knockdown of HIMF expression inhibited this process. In the TAC-induced mouse model, ablation of the HIMF gene attenuated TAC-induced cardiac hypertrophy and fibrosis, and improved cardiac function. The activation of HIF-1α and Ca^2+^-activated calcineurin (CaN)–nuclear factor of activated T cell (NFAT), as well as the MAPK pathway, contributed to the hypertrophic growth of cardiomyocytes. Interestingly, HIMF is not expressed by cardiac fibroblasts. However, the extracellular application of recombinant HIMF protein and conditioned medium from cultured cardiomyocytes overexpressing HIMF still induced fibroblast proliferation, differentiation, and migration. This indicates that HIMF produced in cardiomyocytes during the development of cardiac hypertrophy activates adjacent cardiac fibroblasts via paracrine signaling, inducing fibrosis (Kumar et al., 2019). Another previous study also demonstrated that serum levels of resistin, the human homolog of HIMF, are positively correlated with the severity of heart failure and the risk of adverse cardiac events in patients with heart failure (Zhang et al., 2011). While HIMF is not expressed in humans, the effects of HIMF on the development of cardiac hypertrophy and deterioration of cardiac function in mice may serve as a model for the function of resistin in the human heart under pathological conditions.

#### Myocardial Infarction

Recently, HIMF has been found to be involved in inhibiting apoptosis and increasing proliferation in cardiac fibroblasts, which result in cardiac fibrosis in a myocardial infarction (MI) mouse model. Furthermore, HIMF knockout conferred cardioprotective features on mice, with the suppression of cardiomyocyte apoptosis, fibroblast proliferation (Kim et al., 2021). These are contributed by adiponectin-enriched unsorted bone marrow cells (UBCs). Similarly, HIMF deficiency has also been found to reduce myocardial infarct size and improve cardiac function after MI (Li et al., 2021). HIMF overexpression also directly increased CHOP expression in BMDM and RAW264.7 cells, and regulated macrophage polarization (Li et al., 2021). Taken together, these studies suggest a complex role for HIMF during cardiac injury.

#### Atherosclerosis

HIMF increases EC and VSMC proliferation and, thus, angiogenesis. In addition, HIMF also induces vascular inflammation, which increases vascular permeability and cell adhesion to the endothelium. These functions suggest HIMF may also participate in the development and progression of atherosclerosis, although this has not been confirmed *in vivo*. However, resistin and RELMβ, the two human homologs of HIMF, have been shown to play critical roles in atherosclerosis. Resistin is expressed in atherosclerotic plaque in patients (Jurin et al., 2018; Liberale et al., 2018), and its plasma level is linked to both coronary heart disease (Pourmoghaddas et al., 2020) and future cardiovascular-associated mortality (Park et al., 2017). At the cellular level, resistin upregulates the expression of inflammatory cytokines and adhesion molecules in human ECs (Macdonald et al., 2017), induces SMC proliferation and migration, and promotes foam cell formation (Park et al., 2017). *In vivo* experiments on rabbits have also provided direct evidence that resistin aggravates atherosclerosis by stimulating monocytes, endothelial cells, and vascular smooth muscle cells, inducing vascular inflammation (Cho et al., 2011). Another previous study has also revealed the involvement of RELMβ in atherosclerotic progression (Kushiyama et al., 2013). RELMβ was abundantly expressed in foam cells within plaques from human samples. Furthermore, ablation of RELMβ significantly reduced lipid accumulation in the aortic root and wall in apolipoprotein E-deficient mice, and the inflammatory response of primary cultured peritoneal macrophages. This indicated that RELMβ contributes to atherosclerosis development via lipid accumulation and inflammatory facilitation. RELMβ has also previously been reported to contribute to the regulation of local immune responses in both gut (Ahmed et al., 2019) and bronchial epithelial cells (Fang et al., 2012). The discovery that it is also expressed in foam cells suggests that the tissue-specific distribution of RELM family members may not be particularly strict, and that these proteins can be expressed in unexpected tissues under pathological conditions.

### Metabolic Disorders

The prolonged intraperitoneal administration of HIMF has been found to significantly increase insulin resistance in mice, but the mechanisms remain unknown (Al-Azzawi et al., 2007). Chronic low-grade inflammation is associated with metabolic complications associated during obesity, including insulin resistance. HIMF was readily detected in the serum at baseline, and its level was regulated by energy uptake, strongly suggesting that HIMF has a metabolic role. A previous study in a murine model of dextran sodium sulfate (DSS)-induced colitis and glucose disorder suggested that inflammation may be the mechanism underlying HIMF-induced dysregulation of glucose metabolism and energy balance (Munitz et al., 2009). HIMF serum levels were upregulated in DSS-induced colitis, and ablation of HIMF both decreased inflammation *in situ* and ameliorated DSS-induced colitis. The suppression of the inflammatory response leads to restoration of glucose tolerance, which is impaired in DSS-induced colitis, and protects the mice from hyperglycemia induced by glucose challenge. It support that HIMF contributes to glucose metabolism when it is induced during the setting of specific intestinal inflammatory conditions and the host is exposed to increased pro-inflammatory cytokines and high glucose intake.

While the lack of metabolic phenotype reported in HIMF knockout mice under steady-state conditions, these animals seem to have developed a compensatory mechanism to maintain normal metabolic homeostasis, as they maintain lower leptin concentrations in the sera despite their normal body weight and normal weight gain upon HFD feeding. Leptin plays an important role in regulating energy intake and expenditure, and has a pro-inflammatory role in colitis. The effect of HIMF to regulate leptin levels may also contribute to its overall pro-inflammatory role *in vivo*.

As HIMF serum levels increased in low-density lipoprotein receptor-deficient mice fed a high fat diet, HIMF exerted a favorable cholesterol-lowering effect and conferred protection against atherosclerosis by increasing cholesterol excretion as bile acids (Lee et al., 2014). As demonstrated in a recent study (Kumamoto et al., 2016), a subset of CD11b^+^CD11c^+^CD11c^+^MHCII^+^ mononuclear phagocytes (MNP) characterized by the expression of CD301b (CD301b^+^), participate in the maintenance of normal glucose metabolism and energy balance through HIMF secretion. Deletion of CD301b^+^ MNPs *in vivo* leads to reduced HIMF expression, significant weight loss and increased insulin sensitivity. Reconstitution of HIMF expression in CD301b^+^ MNP-ablated mice restores body weight and serum glucose level, and maintains the whole body metabolism. How HIMF secreted by CD301b^+^ MNP controls feeding behavior and energy balance remains to be determined. These results suggest that the beneficial and detrimental roles of HIMF in inflammatory diseases are likely influenced by the type of immune stimulus, the duration of the stimulus exposure and the tissue type. However, the exact mechanisms underlying the involvement of HIMF in metabolic diseases remain to be clarified in full.

## Conclusion and Perspectives

As a secreted protein, HIMF exerts potent pro-inflammatory effects, is enriched at sites of inflammation, and its expression is correlated with markers of inflammatory disease. In recent years, the functional diversity of RELM proteins, such as HIMF, has generated increased attention. Mice are the main animal model used to manipulate the expression of these genes and to study their function. However, this also represents the biggest challenge faced when researching the role of RELM proteins and resistin in the pathogenesis of disease. The expression patterns of these proteins differ between humans and rodents; indeed, humans do not express HIMF at all. However, studies in mice do provide a solid starting point when investigating the function of RELM proteins in humans. HIMF has been suggested to play functional roles similar to those of human resistin, due to their similar expression patterns (Nair et al., 2006). If this is the case, the involvement of HIMF in the development of cardiovascular diseases and metabolic disorders in mice may be comparable to that of resistin in human disease. Using HIMF knockout mice could provide useful data that might indicate the role of resistin in these diseases. However, the situation may be different in the respiratory system. Here, HIMF appears to serve similar roles as human RELM beta: namely, inducing vascular remodeling and pulmonary fibrosis in pulmonary diseases, particularly in PH. Therefore, HIMF data obtained in mice should be cautiously translated to human diseases, depending on the tissue localization and pathological context.

Early research has primarily focused on the involvement of HIMF in pulmonary vasoconstriction, vascular remodeling, pulmonary inflammation and angiogenesis, which are key pathophysiological processes that occur during the development of PH. HIMF has also been reported to be involved in the development of lung maturation, pulmonary fibrosis, acute lung injury and bronchial asthma. The expression and distribution of HIMF could contribute to the initiation of diseases via multiple signaling pathways, which may cross-talk with each other in the development of pulmonary hypertension. A growing body of evidence also indicates a significant role for HIMF in lung disease. However, the underlying molecular mechanisms remain unclear in both pulmonary hypertension and other diseases. Additional studies are required to provide a more definitive account. Alternatively, whether HIMF is involved in other lung diseases, such as lung cancer, pneumonia, or chronic obstructive pulmonary disease (COPD) with emphysema, also warrants further investigation. A more thorough understanding of these mechanisms will be necessary in order to characterize HIMF, and its human homologs, as specific targets for the treatment of lung disease.

More recently, the study of RELM proteins in physiology and disease has expanded to include cardiovascular diseases. Clinical investigations and experimental studies have identified a positive correlation between circulating levels of resistin and the risk of MI. In mice, HIMF deficiency is known to facilitate M2 macrophage transformation and to increase collagen formation and fibrin deposition in the infarct region, preventing the myocardial wall from expansion and rupture. This helps preserve contractility after acute MI (Li et al., 2021). However, HIMF also induces cardiac fibroblast proliferation, migration and differentiation during cardiac hypertrophy and heart failure (Kumar et al., 2019). Additionally, HIMF may serve pathogenic functions in hypercholesterolemia (Lee et al., 2014) and immune-mediated liver injury (Pai and Njoku, 2021). Thus, future studies on the pathogenic functions of HIMF should be extended to other pathological conditions. The main details of the studies focusing on the role of HIMF in diseases are demonstrated in Table 1.

**TABLE 1:** Presentation of the studied diseases relating to HIMF.

Author/study	Subjects	Pathological condition	Effects
Teng et al. (2003)	Rat pulmonary microvascular smooth muscle cell	Pulmonary hypertension	HIMF has angiogenic and vasoconstrictive properties by upregulating VEGF production and promotes the proliferation and migration of PSMCs.
Su et al. (2017)	Murine myoblastic cell lines (C2C12 and G7); Human circulating EPCs; Male nude mice	Pulmonary hypertension	HIMF increased IL-18 production in myoblasts and promoted tube formation of the endothelial progenitor cells.
Angelini et al. (2013)	Adult C57BL/6 male mice	Pulmonary hypertension	Pulmonary vascular remodeling in mice induced by chronic hypoxia or antigen challenge is associated with marked increases in HIMF expression.
Liu et al. (2009)	Lung fibroblasts; FX knockout C57BL/6 mice	Pulmonary hypertension	Notch1 signaling in response to HIMF plays a significant role in myofibroblast differentiation during lung fibrosis.
Holcomb et al. (2000)	Rat embryonic dorsal root ganglion (DRG) neurons; BALB/c female mice	Allergic asthma	HIMF inhibited the nerve growth factor (NGF)-mediated survival of rat embryonic dorsal root ganglion (DRG) neurons and NGF-induced CGRP gene expression in adult rat DRG neurons. HIMF may modulate the function of neurons innervating the bronchial tree, and thus alter the local tissue response to allergic pulmonary inflammation.
Yamaji-Kegan et al. (2010)	IL-4 and STAT6 knockout C57BL/6 male mice; Mouse pulmonary microvascular endothelial cells (PMVECs)	Lung inflammation	IL-4 signaling may play a significant role in HIMF-induced lung inflammation and vascular remodeling.
Stutz et al. (2003)	BALB/c female mice; BMnot cell line	Allergic asthma	STAT6 directly regulates IL-4- and IL-13-triggered induction of HIMF expression at the transcriptional level by cooperation with C/EBP.
Fan et al. (2015)	RELMa knockout BALB/c male Mice	Allergic asthma	The expression of HIMF increased typically in OVA-induced pulmonary inflammation and vascular remodeling.
Kumar et al. (2018)	HIMF knockout C57BL/6 male mice; Neonatal ratventricular myocytes	Cardiac hypertrophy	HIMF has a critical role in the development of cardiac Hypertrophy via calcium-dependent and HIF-1α Mechanisms.
Kumar et al. (2019)	HIMF knockout C57BL/6 male mice; Neonatal rat ventricular myocytes and Fibroblasts	Cardiac hypertrophy	IL-6 plays a central role in HIMF-induced cardiac hypertrophy and fibrosis that is mediated by activating the MAPK and CaMKII-STAT3 pathways.
Al-Azzawi et al. (2007)	C57BL/6J lean non-diabetic female mice	Metabolic disorders	HIMF increases insulin resistance and reduces gallbladder optimal tension.
Munitz et al. (2009)	*Retnla*^–/–^ male and female mice (backcrossed to C57BL/6 or BALB/c)	DSS-induced colitis	HIMF deficiency reduced the colitis-induced systemic inflammatory response to protect mouse from hyperglycemia induced by glucose injections.
Kumamoto et al. (2016)	Mgl2-DTR (*Mg*/*2* + ^*^*GFP*^*) C57BL/6 male mice	Metabolic disorders	Reconstituting HIMF in CD301b^+^ MNP-depleted animals restored body weight and normoglycemia.
Lee et al. (2014)	*Ldlr*^–/–^, *Ldlr*^–/–^ *Retnla*^–/–^, *Ldlr*^–/–^ *Retnla-Tg* C57BL/6J male and female mice	Hyperlipidemic and atherosclerosis	HIMF exerts a favorable cholesterol-lowering effect and protects against atherosclerosis by enhancing cholesterol excretion in the form of bile acids in *Ldlr*^–^*^/^*^–^mice.

At present, the receptors for resistin and RELM proteins are not known. A recent study revealed a non-classical receptor for HIMF, CaSR, which mediates intracellular but not extracellular HIMF signal (Zeng et al., 2017). A synthesized membrane-permeable peptide designed to flank the binding domain of CaSR for HIMF significantly attenuated hypoxia-induced PH progression. The therapeutic effect of this peptide on PH is quite inspiring, and there may be potential for its use to be extended to other HIMF-associated diseases. However, future studies focused on discovering the receptors and associated signaling pathways of RELM proteins will be required to fully understand its translational medical role in human diseases.

## Author Contributions

ML and WL prepared the figures. WL wrote the manuscript. Both authors contributed to the article and approved the submitted version.

## Conflict of Interest

The authors declare that the research was conducted in the absence of any commercial or financial relationships that could be construed as a potential conflict of interest.
